# *Drosophila melanogaster* Sperm under Simulated Microgravity and a Hypomagnetic Field: Motility and Cell Respiration

**DOI:** 10.3390/ijms21175985

**Published:** 2020-08-20

**Authors:** Irina V. Ogneva, Maria A. Usik, Maria V. Burtseva, Nikolay S. Biryukov, Yuliya S. Zhdankina, Vladimir N. Sychev, Oleg I. Orlov

**Affiliations:** 1 Cell Biophysics Laboratory, State Scientific Center of the Russian Federation Institute of Biomedical Problems of the Russian Academy of Sciences, 76a, Khoroshevskoyoe shosse, 123007 Moscow, Russia; usik.maria@mail.ru (M.A.U.); burtsevamaria@mail.ru (M.V.B.); lboy90@yandex.ru (N.S.B.); juliaszd@yandex.ru (Y.S.Z.); vnsychev@imbp.ru (V.N.S.); olegtm@bk.ru (O.I.O.); 2 Department of Medical and Biological Physics, I. M. Sechenov First Moscow State Medical University, 8-2 Trubetskaya St., 119991 Moscow, Russia

**Keywords:** sperm, hypomagnetic field, simulated microgravity, motility, cell respiration

## Abstract

The role of the Earth’s gravitational and magnetic fields in the evolution and maintenance of normal processes of various animal species remains unclear. The aim of this work was to determine the effect of simulated microgravity and hypomagnetic conditions for 1, 3, and 6 h on the sperm motility of the fruit fly *Drosophila melanogaster*. In addition to the usual diet, the groups were administered oral essential phospholipids at a dosage of 500 mg/kg in medium. The speed of the sperm tails was determined by video recording and analysis of the obtained video files, protein content by western blotting, and cell respiration by polarography. The results indicated an increase in the speed of movement of the sperm tails after 6 h in simulated microgravity. The levels of proteins that form the axoneme of the sperm tail did not change, but cellular respiration was altered. A similar effect occurred with the administration of essential phospholipids. These results may be due to a change in the level of phosphorylation of motor proteins. Exposure to hypomagnetic conditions led to a decrease in motility after 6 h against a background of a decrease in the rate of cellular respiration due to complex I of the respiratory chain. This effect was not observed in the flies that received essential phospholipids. However, after 1 h under hypomagnetic conditions, the rate of cellular respiration also increased due to complex I, including that in the sperm of flies receiving essential phospholipids.

## 1. Introduction

The prospect of human exploration of deep space indicates the need to answer questions related to the role of various physical factors in the development and maintenance of the viability of the species. In addition to gravity, the electromagnetic field, particularly its magnetic component (the Earth’s magnetic field, a geomagnetic field), is another permanent factor. Nevertheless, the role of these physical fields in the evolution of various animal species and their biological activity remains obscure, which can be due to technical difficulties associated with the creation of microgravity and hypomagnetic conditions in model experiments.

Almost all living organisms at different levels of organization are sensitive to changes in gravity, even during embryogenesis. Thus, in amphibians, thickening of the blastocoel and structural changes at the stage of fragmentation and neurulation were observed [1], and in insects, particularly the fruit fly *Drosophila melanogaster*, changes in egg size and expression of various genes at the larval stage were observed [2,3]. In Japanese quail embryos under space flight, there was a lag in the development of the spinal cord, in mass gain, and in an increase in body size [4,5]. In rats in which the last quarter or half of the prenatal development occurred during space flight, and there was also a lag in mass gain and ossification of the skeleton, which showed rapid compensation on the first day on Earth [6]. However, in animals in the flight group, accelerated development of the thyroid and parathyroid glands, a decrease in the rate of cell division in the thymus, and changes in the nervous tissue with a decrease in the number of neurons in the brain and spinal cord were observed [7,8].

The consequences described above could be caused by changes in various types of cells under microgravity, particularly changes in the cytoskeleton [9,10,11,12,13,14,15], including those involved in the formation of the contractile ring and spindle division, and a decrease in the differentiation potential [16,17]. Moreover, similar changes occur in germ cells, which can be especially significant for the subsequent formation and development of the embryo. In space flight and in model experiments on Earth, the number of mature sperm cells decreased in rodents [18,19,20,21], and changes in their mobility, the structure of the cytoskeleton and the mRNA levels of various genes were observed, including changes in the epigenetic regulation of expression [22,23,24,25].

Thus, from an evolutionary point of view, the influence of gravity on the structural and functional characteristics of germ cells can play a key role in early development and, in general, in maintaining the species. Therefore, one target of changes in gravity at the cellular level is the cytoskeleton, which, inter alia, provides sperm motility and, therefore, its fertilizing ability. However, the energy supply of this motility may be targeted by changes in the electromagnetic field, particularly its magnetic component.

Magnetosensitivity can be evolutionarily determined in marine animals because they are in high conductivity salt water, as well as in animals that have magnetite crystals in their bodies, which allows them to navigate in space [26,27]. In addition, the formation of radical pairs that occur in photoreception, such as that in birds, can also be a target of a magnetic field [28,29]. Therefore, one of the main candidates for the role of magnetosensitive proteins is cryptochromes, photosensitive proteins, which are conserved in a whole series of taxa and allow cells to perceive blue and ultraviolet light. In experiments using Cry-null mutants of the fruit fly *D. melanogaster*, negative geotaxis through a Cry-dependent mechanism was reduced in a field with a magnetic induction of 500 μT [30].

The magnitude of the Earth’s magnetic field, depending on latitude, is 25–65 μT. Therefore, one would expect that a decrease in the magnitude of the magnetic induction would not have significant effects. However, the results of a few experimental studies, mainly using Helmholtz rings, indicated that a field decrease of approximately 1000 times (up to 10 nT at a frequency of 50 Hz) after 3 days of exposure leads to a change in the behavioral characteristics of mice and an increase in anxiety [31]. Moreover, in similar experiments with *D. melanogaster*, changes in learning ability, up to complete amnesia, were noted even 10 generations after a 24-min exposure in a field of 100–680 nT [32]. In addition, shielding the geomagnetic field to a magnetic induction of less than 200 nT and exposing C57Bl/6 mice stimulated an increase in markers of neurogenic differentiation and increased in the number of proliferating cells in the subventricular zone of the brain [33]. A slightly smaller shielding of the field (up to 0.2 μT) led to abortion of early mouse embryos, although changes in development were not documented before the blastocyst stage [34]. According to the authors, hatching from the zona pellucida, implantation and subsequent interaction of the trophoblast with the endometrium could be changed in a hypomagnetic field [34].

Considering that the magnetic field acts on moving electric charges and bodies with a magnetic moment, it can be assumed that the main targets, in addition to the components of photoreception, will be electrons moving along the respiratory chain of mitochondria and ions moving along ion channels.

The fruit fly *D. melanogaster* is a convenient model for exposing the whole organism during research under both microgravity and hypomagnetic conditions in model experiments on Earth due to its convenient handling, short life cycle, and well-studied physiology and genetics.

Therefore, in this work, we evaluated the motility and cytoskeleton state, cell respiration, and state of the respiratory chain of the sperm of the fruit fly *D. melanogaster* under simulated microgravity or hypomagnetic field conditions for 1, 3, and 6 h, with or without treatment with essential phospholipids, which can modulate the structure of the actin cytoskeleton and the expression of the components of the respiratory chain.

## 2. Results

### 2.1. Sperm Motility

**Figure 1:**
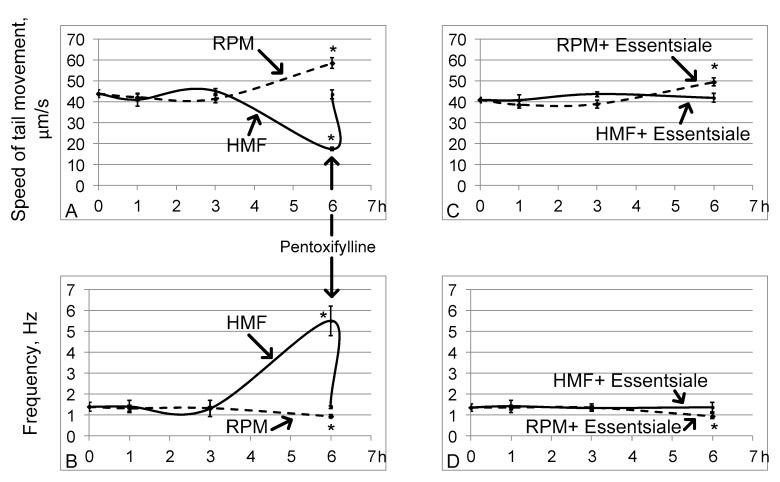
The speed of movement of the end of the tail of the sperm and its frequency. The solid line shows the changes in hypomagnetic conditions, and the dashed line shows the microgravity simulation using random position machine. * *p* < 0.05 compared with the control group C. (**A**,**B**) are the speed of movement and frequency, respectively, without oral administration of essential phospholipids. The arrow shows that the addition of pentoxifylline at a dilution of 1:2.5 leads to an increase in the speed of movement and a decrease in the frequency of beating of the end of the tail of the sperm after a 6-h exposure under hypomagnetic conditions. (**C**,**D**) are similar parameters (as in (**A**,**B**)) but with the oral administration of essential phospholipids.

**Figure 2:**
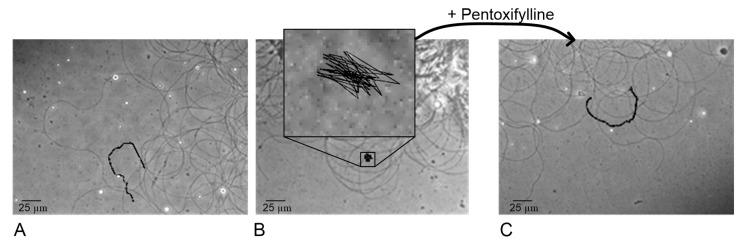
Tracking the end of the tail of the sperm in the control group C (**A**), group HMF6 after 6 h of exposure under hypomagnetic conditions (**B**) and the group after addition of pentoxifylline to this sample at a dilution of 1: 2.5 (**C**).

The linear speed of the end of the sperm isolated from the seminal vesicles after 1 and 3 h of exposure to hypomagnetic conditions (groups HMF1 and HMF3) did not change relative to that in the control group (group C) (Figure 1A). However, after 6 h, this speed decreased by 60% (*p* < 0.05) compared to that of group C (17.4 ± 0.4 μm/s in the HMF6 group vs. 43.8 ± 1.8 μm/s in group C) (Figure 1A). Moreover, the frequency of movement of the end of the sperm increased sharply, more than 3 times relative to that of the control (5.5 ± 0.7 Hz in the HMF6 group vs. 1.39 ± 0.21 Hz in group C) (Figure 1B), which is reflected in the trajectory, describing the end of the sperm (Figure 2). The addition of pentoxifylline to a solution with sperm of the flies of the HMF6 group at a dilution of 1:2.5 significantly increased the speed of movement (to 43.6 ± 2.1 μm/s) and reduced the frequency (to 1.37 ± 0.07 Hz) to the control level (Figure 1A,B).

Exposing the fruit flies to simulated microgravity conditions led to the opposite effect: after 6 h (in the RPM6 group), the speed increased by 34% (*p* < 0.05), and the frequency decreased by 33% (*p* < 0.05), amounting to 58.5 ± 2.6 μm/s and 0.93 ± 0.09 Hz, respectively (Figure 1A,B). In this case, changes in motility in groups RPM1 and RPM3 relative to group C, as well as in hypomagnetic conditions, were not observed.

Oral administration of essential phospholipids in the control group did not change the linear speed of the tail end of the sperm (43.8 ± 1.8 μm/s in group C and 40.9 ± 1.3 μm/s in group CE) or its frequency of movement (1.39 ± 0.21 Hz in group C and 1.35 ± 0.17 Hz in the CE group). Furthermore, essential phospholipids prevented a decrease in the speed and an increase in the frequency of movement of the end of the sperm after 6 h in hypomagnetic conditions (group HMFE6) (Figure 1C,D). However, an increase in the speed of movement of the end of the tail of the sperm and a decrease in the frequency of its beating after 6 h of simulated microgravity occurred with treatment of essential phospholipids (group RPME6) (Figure 1C,D).

### 2.2. Relative Protein Content

**Figure 3:**
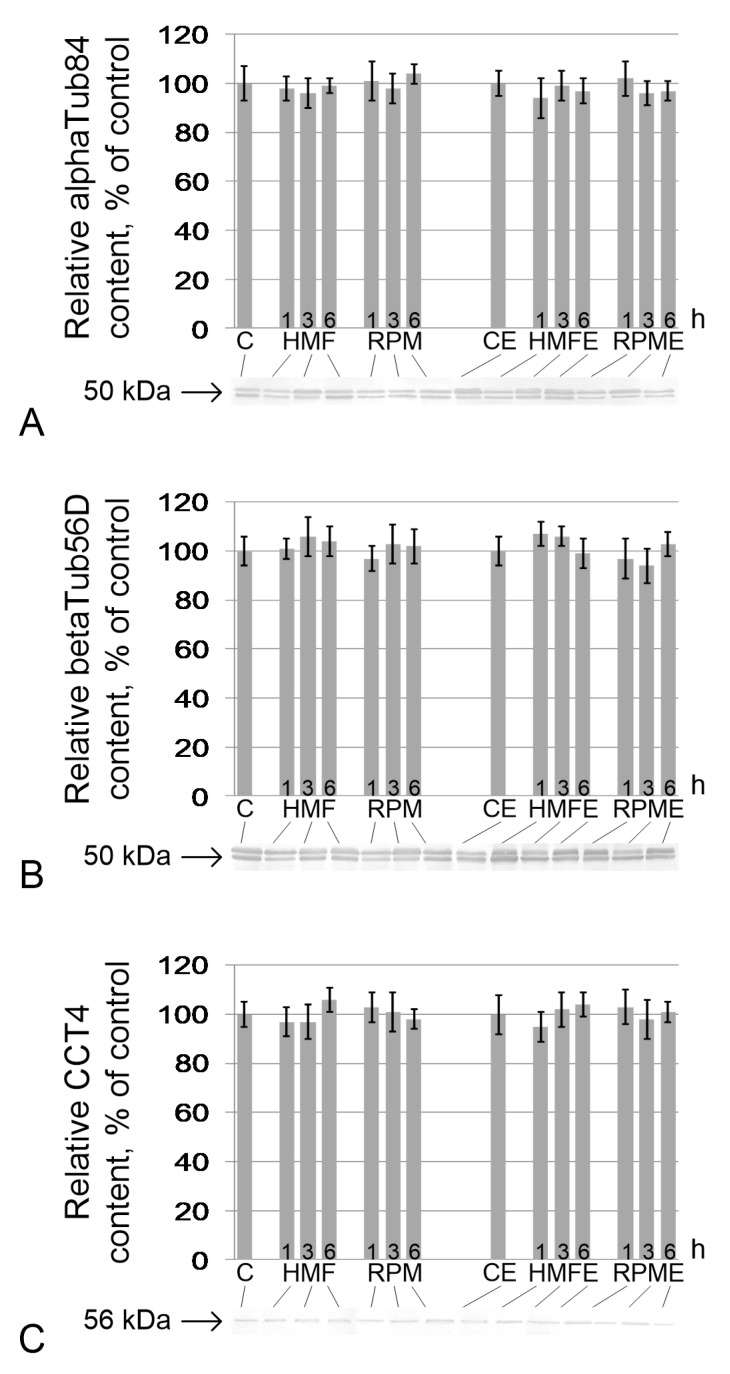
Relative content of proteins and components of the sperm tail axoneme. (**A**) alphaTub84, alpha-tubulin84D, and alpha-tubulin84B (50 kDa), components of the tubulin heterodimer; (**B**) betaTub56D, beta-tubulin (50 kDa), component of the tubulin heterodimer; (**C**) Cct4 (CG5525), chaperonin containing Tcp1 subunit 4-delta (56 kDa), participates in the assembly of tubulin heterodimers. Typical western blots for each protein are performed under the according histogram.

The relative contents of alpha-tubulin (Figure 3A), beta-tubulin (Figure 3B), and subunit 4 of the Tcp1 complex (Figure 3C), forming the axoneme of the tail of the sperm, did not change relative to the control contents both under hypomagnetic conditions and in simulated microgravity. There were also no changes in the groups receiving essential phospholipids (Figure 3).

**Figure 4:**
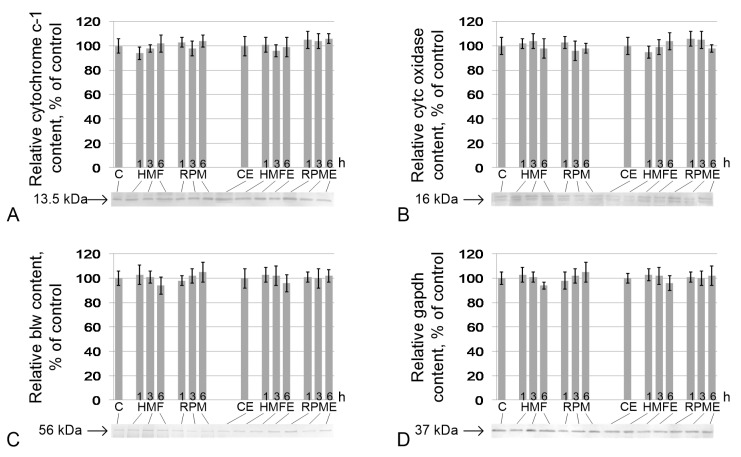
Relative contents of proteins that participate in cell respiration. (**A**) cytochrome *c*-1 (CG4769) (13.5 kDa), protein of the respiratory chain, located between complexes III and IV; (**B**) cytochrome *c* oxidase (CG10396) (16 kDa), protein of complex IV of the respiratory chain; (**C**) blw (bellwether), ATP synthase F1 (56 kDa), subunit of ATP synthase; (**D**) gapdh, glyceraldehyde-3-phosphate dehydrogenase (37 kDa), catalyzes one step of the glycolytic breakdown of glucose. Typical western blots for each protein are shown under the according histogram.

Similarly, no changes were noted in the content of proteins involved in ATP generation. There were no changes in the relative contents of cytochrome *c* (Figure 4A), cytochrome c oxidase (Figure 4B), blw (the catalytic subunit F1 ATP synthase) (Figure 4C), and gapdh (Figure 4D) in the HMF and RPM groups, as well as in HMFE and RPME up to 6 h of exposure.

### 2.3. Cell Respiration

The rates of oxygen absorption by the testes of the flies of groups C and CE did not significantly differ from each other.

**Figure 5:**
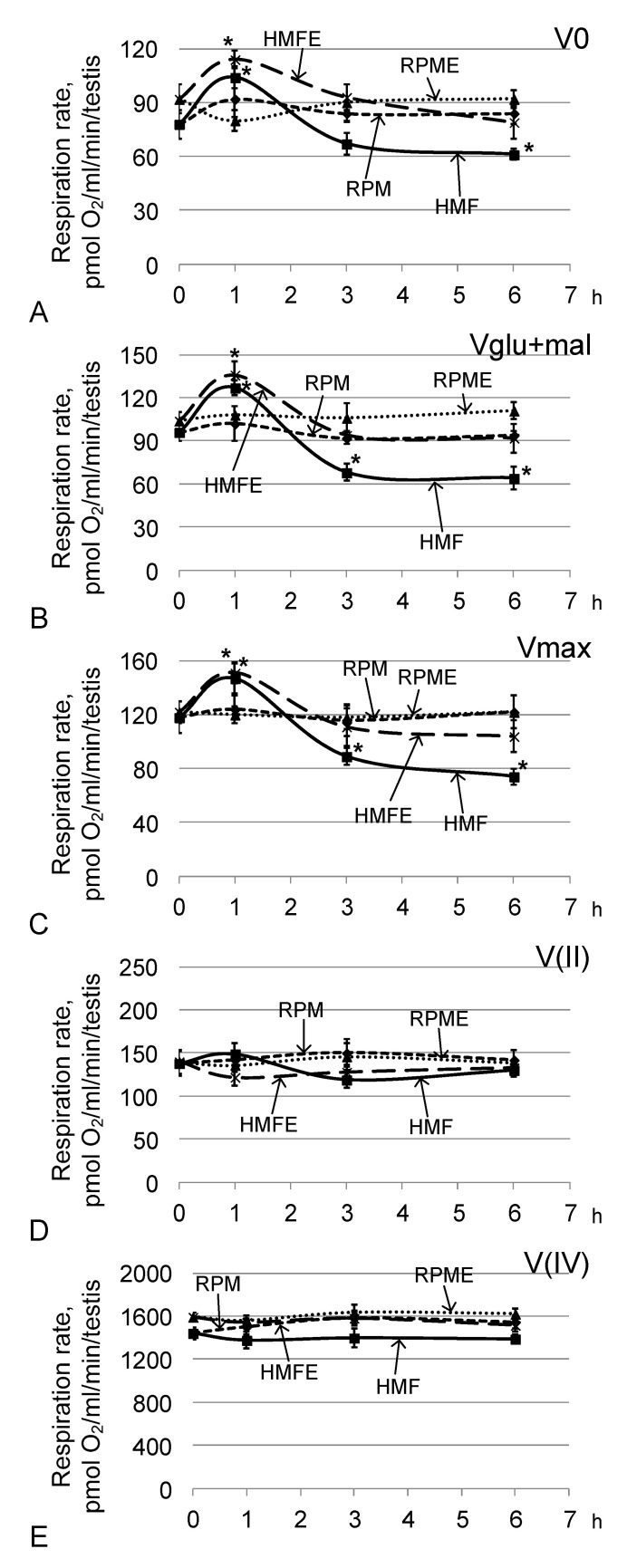
Testis respiration rate after fly exposure under hypomagnetic and microgravity conditions. (**A**) V0, respiration rate of permeabilized cells; (**B**) Vglu + mal, respiration rate after adding 10 mM glutamate + 5 mM malate; (**C**) Vmax, maximum respiration rate after adding 2 mM ADP; (**D**) V(II), respiration rate after adding 0.5 μM rotenone (complex I inhibitor) and the subsequent supplement 10 mM succinate (substrate of complex II); (**E**) V(IV), respiration rate after adding 5 μM antimycin (complex III inhibitor) and the subsequent supplement 0.5 mM TMPD + 2 mM ascorbate (artificial substrates of complex IV). * *p* < 0.05 compared with the corresponding control group.

Under hypomagnetic conditions, after 1 h of exposure, the cell respiration rate increased (Figure 5). In groups HMF1 (relative to group C) and HMFE1 (relative to group CE), V0 (Figure 5A) was higher by 33% and 24% (*p* < 0.05), respectively; Vglu + mal (Figure 5B) was higher by 32% and 31% (*p* < 0.05), respectively; and Vmax (Figure 5C) was higher by 25% and 24% (*p* < 0.05), respectively. After 3 h, the respiratory rate with the addition of substrates of the respiratory chain complex I (Vglu + mal) (Figure 5B) and the maximum speed (Vmax) (Figure 5C) in the HMF3 group decreased by 29% (*p* < 0.05) and 25% (*p* < 0.05), respectively, compared with those of group C. After 6 h, this decrease was more pronounced: V0 was decreased by 22% (*p* < 0.05), Vglu + mal by 33% (*p* < 0.05), and Vmax by 37% (*p* < 0.05) relative to similar indicators in group C. However, in the groups receiving essential phospholipids, none of the indicators of cell respiration differed from the controls, either after 3 h or after 6 h.

The oxygen absorption rates by permeabilized testes (V0) (Figure 5A), the absorption rate upon addition of exogenous substrates (Vglu + mal) (Figure 5B), the maximum rate upon addition of a saturating concentration of ADP (Vmax) (Figure 5C), and the respiration rate after inhibition of the first complex of the respiratory chain V (II) (Figure 5D) and the third complex of V (IV) (Figure 5E) in the RPM group and the RPME group did not differ from the corresponding control levels.

## 3. Discussion

The interaction of cells and various physical fields is still one of the least studied problems of biophysics. It is still unclear how the gravitational and magnetic fields of the Earth affect the structural and functional characteristics of various types of cells. In this work, we analyzed the sperm of the fruit fly *D. melanogaster* given the convenience of working with this model when simulating microgravity and hypomagnetic conditions. Very short exposure times were chosen to identify the earliest events of the cell response to changes in external physical conditions. Sperm motility was analyzed as a clear marker for structural and functional changes. Due to the structural features (very long and thin tails), isolation of freely moving spermatozoa of *D. melanogaster* has multiple technological difficulties [35]. Usually, they are isolated from the female genital tract, but in our experiment, the exposure was up to 6 h, which makes such a technique impossible for our purposes. Therefore, we analyzed sperm obtained by dissection of seminal vesicles that were partially associated with the tissue of the testes.

### 3.1. Simulated Microgravity

The linear speed of the end of the tail of the sperm of *D. melanogaster* increased against the background of a decrease in its beating frequency, which under physiological conditions with free movement in the female genital tract would increase the progressive movement. We observed this effect after 6 h of exposure under simulated microgravity conditions. Moreover, the contents of proteins that form the axoneme of the tail of the sperm did not change. There were also no changes in the rate of cell respiration in any of the study groups.

Notably, in experiments in real space flight, the motor activity of the sea urchin spermatozoa increased significantly, which is probably due to an increase in the phosphorylation level of phosphothreonine- and phosphoserine-containing proteins [36,37]. In contrast, in mammals, the speed of sperm movement decreased. This result has been shown in experiments with animals that underwent antiorthostatic suspension [22,38] and during exposure of isolated sperm of humans and mice to simulated microgravity conditions [39,40].

This interspecific difference, due to the high conservatism of the proteins that form the axoneme of the sperm tail, is most likely due to various regulatory mechanisms of phosphorylation of motor proteins. For sea urchin spermatozoa, the increased the speed of movement was apparently associated with phosphatases, since inhibition of protein kinases did not affect the observed effect [37]. However, similar data for mammals were not found in the available literature.

In addition to the terminal stages of phosphorylation regulation, the question of triggering the signaling pathway remains. We previously suggested that cell mechanoreception can be realized through deformation of the membrane and cortical cytoskeleton, leading to the initiation of various signaling pathways with increases and decreases in external mechanical stress [41,42]. Moreover, a change in the lipid composition of membranes and a decrease in cholesterol, which leads to rearrangements of the cortical cytoskeleton and an increase in the resistance of these structures to deformations, allowed us to prevent the effects of simulated microgravity in various cases, including for mouse sperm [22,43]. However, in this study, even with the use of essential phospholipids, the increased linear speed of the tail of the sperm of the fruit fly was maintained, although it was not as pronounced. This result may be due to the characteristics of the metabolism of insects that are cannot synthesize cholesterol [44], the predominance of phosphoethanolamine in the cell membranes [45] and, probably, another mechanism that regulates the anchoring of the submembrane cytoskeleton to the membrane. The administration of additional essential phospholipids probably led to some rearrangements and to a subsequent decrease in the increased speed, but not its prevention, although these results were not as significant as those in mammals.

### 3.2. Hypomagnetic Conditions

Unlike simulated microgravity, under hypomagnetic conditions, the speed of movement of the end of the tail of the sperm decreased significantly, and the frequency of beating increased after 6 h of exposure. Moreover, this effect can be offset by the addition of pentoxifylline. Pentoxifylline, a phosphodiesterase inhibitor, increases cAMP concentration [46,47] and tyrosine phosphorylation [48,49]. In addition, the oral administration of essential phospholipids prevented a decrease in speed and an increase in the frequency of beating of the tail of the sperm. In a cell, cAMP is synthesized by adenylate cyclase from ATP. Since the target of the magnetic component of the electromagnetic field is moving electric charges, such as electrons in the respiratory chain of mitochondria, we hypothesized that a decrease in ATP production may be the reason for the decrease in motility under hypomagnetic conditions. Therefore, we measured the rate of cell respiration.

Indeed, after 6 h, the maximum respiration rate decreased. Moreover, after 1 h of exposure, the rate of cell respiration increased, based on the results of the inhibitor analysis, due to complex I of the respiratory chain. The activity of NADH dehydrogenase in this complex is one of the main causes of electron leakage and the formation of reactive oxygen species (ROS) in sperm [50]. In turn, ROS are normally transformed into peroxide, and the accumulation of ROS can lead to lipid peroxidation and an increase in the malondialdehyde content [51]. Malondialdehyde, which inhibits cytochrome *c* oxidase (complex III of the respiratory chain), leads to a decrease in cell respiration. However, such a sequence of events is not suitable for explaining the effect of decreased cellular respiration after 6 h of exposure under hypomagnetic conditions. Blockade of complex I by rotenone and the addition of succinate (substrate of complex II) showed that there are no changes in the rate in this case. Therefore, a decrease in respiration after 6 h, as well as its increase after 1 h, occurred due to complex I of the respiratory chain but not complex III.

Moreover, in the group receiving essential phospholipids, after 6 h in hypomagnetic conditions, no changes in speed were observed in any of the sections of the respiratory chain, which is consistent with the absence of a change in motility. However, an increase in the respiration rate after 1 h of exposure under hypomagnetic conditions was observed in this case as well and due to complex I of the respiratory chain. Moreover, we expected that the use of essential phospholipids would enhance the negative effect due to the predominant oxidation of unsaturated fatty acids. However, the effect of decreased respiration and motility after 6 h was absent.

Thus, the key question of changing the efficiency of complex I of the respiratory chain in mitochondria under hypomagnetic conditions remains. These results may be due to a change in its redox potential. A 20–30% change in respiration efficiency may be associated with a small change in the potential of complex I and, with an even smaller contribution, in general, to the redox potential. Thus, potential-sensitive probe techniques are not sufficiently sensitive.

## 4. Materials and Methods

### 4.1. Experimental Design

Two-day-old virgin males of the *D. melanogaster* Canton S line were placed in 50 mL Falcon tubes (40 animals per tube) with a breathable cap containing 10 mL of *Drosophila* dilution medium (agar—0.7%, sugar—4%, semolina—4%, baker’s yeast—2.5%, propionic acid—1%). Essential phospholipids in the form of EssentsialeR Forte N (A. Nattermann and Cie. GmbH, Köln, Germany) at a dose of 500 mg/kg of medium was added to half the tubes. The administration of essential phospholipids began with the previous generation so that all stages of development occurred on a modified nutrient medium.

Fourteen study groups were formed:

- C: a control group receiving a standard nutrient medium that was kept under standard conditions;
- HMF1: a group receiving a standard nutrient medium that remained under hypomagnetic conditions for 1 h;
- HMF3: a group receiving a standard nutrient medium that remained under hypomagnetic conditions for 3 h;
- HMF6: a group receiving a standard nutrient medium that remained under hypomagnetic conditions for 6 h;
- RPM1: a group receiving a standard nutrient medium that remained under simulated microgravity conditions for 1 h;
- RPM3: a group receiving a standard nutrient medium that remained under simulated microgravity conditions for 3 h;
- RPM6: a group receiving a standard nutrient medium that remained under the simulated microgravity conditions for 6 h;
- CE: a control group receiving a nutrient medium supplemented with essential phospholipids that remained under standard conditions;
- HMFE1: a group receiving a nutrient medium supplemented with essential phospholipids that remained under hypomagnetic conditions for 1 h;
- HMFE3: a group receiving a nutrient medium supplemented with essential phospholipids that remained under hypomagnetic conditions for 3 h;
- HMFE6: a group receiving a nutrient medium supplemented with essential phospholipids that remained under hypomagnetic conditions for 6 h;
- RPME1: a group receiving a nutrient medium with the addition of essential phospholipids that remained under simulated microgravity conditions for 1 h;
- RPME3: a group receiving a nutrient medium supplemented with essential phospholipids that remained under simulated microgravity conditions for 3 h;
- RPME6: a group receiving a nutrient medium supplemented with essential phospholipids that remained under simulated microgravity conditions for 6 h.

Hypomagnetic conditions were created in Helmholtz rings, designed in such a way as to compensate for the magnetic field of the Earth, which in our laboratory is 48 μT. Test tubes with flies were placed in the center of coaxially located radial coils, where a zone with a uniform hypomagnetic field was formed. During the experiment, the magnitude of the magnetic induction vector was 0.000 ± 0.001 μT [52].

Microgravity conditions were created using a random positioning machine (RPM), which provides 3D multidirectional rotation relative to the gravity vector, so that the superposition of the orientation vectors of the objects in the gravitational field is equal to zero per minute on average [53]. Test tubes with flies were placed in the center of the platform and exposed under the same conditions as the control groups.

At the end of the exposure, seminal vesicles were removed from the flies of the experimental and control groups, some of which were used to analyze motility and determine cellular respiration, while the rest were frozen for subsequent isolation of protein.

All the experimental procedures were approved by the Commission on Biomedical Ethics of the Institute of Biomedical Problems (IBMP), the State Scientific Center of the Russian Federation and the Federal State Budgetary Institution of Science (Minutes No. 521 dated 25 September 2019).

### 4.2. Estimation of Sperm Motility

After extirpation, seminal vesicles were dissected in physiological solution to obtain sperm. Then, a drop of the suspension was applied to a Makler chamber (Sefi Medical Instruments, Ltd., Haifa, Israel) and observed in a phase-contrast microscope (Eclipse E200 MV, Nikon, Tokyo, Japan) with a magnification of 200×. For sperm motility analysis, we made video recordings by a Basler puA1600-60uc color camera with an e2V EV76C570 CMOS sensor and 60 frames per second with 2-megapixel resolution (Basler AG, Ahrensburg, Germany).

We calculated the frequency and speed of tail movement as the main parameters of sperm motility. Frequency (in Hz) was estimated as the established line crossing frequency [54]. To calculate the speed (μm/s), we monitored the end of the tail of the sperm, and the distance it traveled per second was measured. Motility data were analyzed in the ImageJ program (https://imagej.nih.gov/ij/) using the plugin Manual Tracking.

### 4.3. Evaluation of Protein Content by Western Blotting

The evaluation of protein content was described in detail previously [40]. Briefly, for protein extraction part of frozen testes from each group of flies was used. Testes were homogenized in Laemmli buffer with a protease inhibitor cocktail (Calbiochem, San Diego, CA, USA). Denaturing electrophoresis on polyacrylamide gels was performed using the Laemmli method (Bio-Rad Laboratories, Hercules, CA, USA). An equal amount of protein was placed into each well (based on the measured concentration), separated by electrophoresis, and transferred to a nitrocellulose membrane [55].

The following specific primary monoclonal antibodies were used at the dilutions recommended by the manufacturers to determine the levels of each protein: mouse antibodies against cytochrome *c*-1 (MW 13.5 kDa, 5 μg/mL, Abcam, Cambridge, UK, #ab13575), cytochrome *c* oxidase (MW 16 kDa, 1 μg/mL, Abcam, UK, #ab14744), ATP synthase F1 (blw) (MW 56 kDa, 1 μg/mL, Abcam, UK, #ab14748), gapdh (MW 37 kDa, diluted 1:1000, Abm, Canada, #G041), rabbit antibodies against alphaTub84 (MW 50 kDa, diluted 1:1000, Abcam, UK, #ab52866), betaTub56D (MW 50 kDa, diluted 1:1000, Abcam, UK, #ab179513), and CCT4 (MW 56kDa, 1.25 μg/mL, Abcam, UK, #ab49151).

As the secondary antibodies to detect mouse IgG we used biotinylated goat antibodies (Sigma, Hamburg, Germany, #B9904) at a dilution of 1:20,000 and to detect rabbit IgG—biotinylated goat antibodies (Jackson ImmunoResearch Lab, Inc., West Grove, PA, USA, #111-035-003) at a dilution of 1:10,000.

For detection, the membranes were treated with streptavidin solution conjugated with horseradish peroxidase (Sigma, Germany, #E2886) at a dilution of 1:10,000, and after it, the protein bands were revealed using 3,3′-diaminobenzidine (Amresco, Solon, OH, USA, #E733-50). Protein bands were analyzed by the ImageJ program.

### 4.4. Estimation of Cell Respiration by Polarography

Twenty testes from each experimental group were used to analyze cell respiration by polarography according to the protocol described in detail Kuznetsov et al. [56]. After isolation of the seminal vesicles and their dissection, the permeabilizing agent saponin at a concentration of 10 μg/mL was added to the medium with physiological solution and incubated for 15 min at +22 °C [56]. Then, the sample was transferred to a polarographic cuvette. Changes in oxygen concentration were measured using Oxygraph+ (Hansatech Instruments, Ltd., Norfolk, UK) at 22 °C.

A substrate-inhibitory assay was conducted according to the protocol of Kuznetsov et al. [56] and our previous study [40]. After the sample was transferred to the polarographic cuvette, we recorded the rate of oxygen uptake by permeabilized cells—V0. Then, we added 10 mM glutamate and 5 mM malate (substrates of the first complex of the respiratory chain) and recorded the respiratory rate Vglu + mal. Next, we added 2 mM ADP and recorded the maximum respiration rate—Vmax. Then, we added inhibitors and substrates of the following respiratory chain complexes to analyze their functional activity: 0.5 μM rotenone (complex I inhibitor), 10 mM succinate (complex II substrate) and recorded oxygen absorption rate V (II); after it, we added 5 μM antimycin A (inhibitor complex III), and 0.5 mM TMPD + 2 mM ascorbate (artificial substrates of complex IV) and recorded the oxygen absorption rate V (IV). The test for intactness of the outer mitochondrial membrane was carried out for each sample by adding 10 μM cytochrome *c* after inhibition analysis: If the membrane is intact, the respiratory rate does not change or increases by a maximum of 15%. The cellular respiration rate is expressed as pmol O_2_ per mL per min pes testis. For each experimental point, we tested at least three biological replicates.

### 4.5. Statistical Analysis

For the statistical analysis of the results obtained ANOVA with the post hoc *t*-test with a significance level of *p* < 0.05 to assess the reliability of differences between the groups was used. We presented our data as the mean ± standard error of the mean (M ± SE).

## 5. Conclusions

In this study, we attempted to analyze the earliest effects of sperm perception of a change in two fundamental physical fields under which evolution occurred. Of course, long flights beyond the Earth’s magnetosphere are a distant future, but to successfully maintain the health of the species, it is necessary to understand how the perception of the gravitational and electromagnetic fields occurs at the cellular level. The results obtained indicate that the motility of the tail of the sperm of *D. melanogaster* increases under simulated microgravity and decreases under hypomagnetic conditions. The absence of changes in the protein pattern indicates subtle mechanisms for regulating the response of cells to changes in physical fields. In the first case, the change is probably associated with the regulation of phosphorylation of motor proteins and, in the second, with a decrease in the energy supply of motility. However, both factors require further study.

Limitations of the study are associated with the choice of *Drosophila melanogaster* as the object of the study. On the one hand, it is a very convenient object for such studies: it is small and so the whole organism could be placed into the homogeneous hypomagnetic field without any fluctuations and it was possible to use a random position machine for microgravity simulation. Moreover, structure and motility regulation of spermatozoa, generally similar to the structure of mammalian sperm. On the other hand, deep space exploration will be implemented by humans and so investigation of mammalian sperm in such conditions is required. But, in turn, it requires another device. So, we suggest that the investigation of *Drosophila melanogaster* could be considered as the first step of the study of the influence of hypomagnetic and microgravity conditions to the sperm.
